# Promoting cross-jurisdictional primary health care research: developing a set of common indicators across 12 community-based primary health care teams in Canada

**DOI:** 10.1017/S1463423618000518

**Published:** 2018-11-06

**Authors:** Sabrina T. Wong, Julia M. Langton, Alan Katz, Martin Fortin, Marshall Godwin, Michael Green, Eva Grunfeld, Kasra Hassani, Claire Kendall, Clare Liddy, Jenny Ploeg, Walter P. Wodchis, Jeannie L. Haggerty

**Affiliations:** 1 Centre for Health Services and Policy Research; and School of Nursing, University of British Columbia, Vancouver, BC, Canada; 2 Centre for Health Services and Policy Research, University of British Columbia, Vancouver, BC, Canada; 3 Departments of Community Health Sciences and Family Medicine, Rady Faculty of Health Sciences, University of Manitoba, Winnipeg, MB, Canada; 4 Département de médecine de famille et de médecine d’urgence, Université de Sherbrooke, Sherbrooke, QC, Canada; 5 Department of Family Medicine, Memorial University of Newfoundland, St. John’s, NL, Canada; 6 Departments of Family Medicine and Public Health Sciences, Queen’s University, Kingston, ON, Canada; 7 Department of Family and Community Medicine, University of Toronto, Toronto, ON, Canada; 8 Ontario Institute for Cancer Research, Toronto, ON, Canada; 9 C.T. Lamont Primary Health Care Research Centre, Bruyère Research Institute, Ottawa, ON, Canada; 10 School of Nursing, Faculty of Health Sciences, McMaster University, Hamilton, ON, Canada; 11 Institute of Health Policy, Management and Evaluation, University of Toronto, Toronto, ON, Canada; 12 Department of Family Medicine, McGill University, Montréal, QC, Canada

**Keywords:** collaboration, common indicators, cross-jurisdictional, innovation, measurement, primary health care

## Abstract

**Aim:**

To describe the process by which the 12 community-based primary health care (CBPHC) research teams worked together and fostered cross-jurisdictional collaboration, including collection of common indicators with the goal of using the same measures and data sources.

**Background:**

A pan-Canadian mechanism for common measurement of the impact of primary care innovations across Canada is lacking. The Canadian Institutes for Health Research and its partners funded 12 teams to conduct research and collaborate on development of a set of commonly collected indicators.

**Methods:**

A working group representing the 12 teams was established. They undertook an iterative process to consider existing primary care indicators identified from the literature and by stakeholders. Indicators were agreed upon with the intention of addressing three objectives across the 12 teams: (1) describing the impact of improving access to CBPHC; (2) examining the impact of alternative models of chronic disease prevention and management in CBPHC; and (3) describing the structures and context that influence the implementation, delivery, cost, and potential for scale-up of CBPHC innovations.

**Findings:**

Nineteen common indicators within the core dimensions of primary care were identified: access, comprehensiveness, coordination, effectiveness, and equity. We also agreed to collect data on health care costs and utilization within each team. Data sources include surveys, health administrative data, interviews, focus groups, and case studies. Collaboration across these teams sets the foundation for a unique opportunity for new knowledge generation, over and above any knowledge developed by any one team. Keys to success are each team’s willingness to engage and commitment to working across teams, funding to support this collaboration, and distributed leadership across the working group. Reaching consensus on collection of common indicators is challenging but achievable.

## Background

Strong primary care systems are associated with better patient outcomes, particularly for those with chronic conditions (Hansen *et al*., 2015). Yet around the globe, primary care clinicians report challenges in coordinating care and delivering care to the most complex and vulnerable patients (Osborn *et al*., 2015). This points to the need for targeted efforts to innovate in the delivery of primary health care with a particular focus on strategies to effectively reach the most vulnerable patients.

Research in primary care plays a critical role in informing, evaluating, and helping improve the delivery and organization of health care services (Hutchison *et al*., 2011; Hutchison and Glazier, 2013). The diversity of primary care activity within and across jurisdictions (eg, provinces, states, countries) presents an opportunity for learning about which innovations could be spread and scaled-up to provide better service to vulnerable populations and patients with chronic diseases. By comparing changes in primary care delivery across geographies and provincial/territorial boundaries, researchers, together with clinicians, decision-makers, and patients, can produce knowledge about what is needed to spread promising innovations across regions and which are reproducible on a larger scale.

A pan-Canadian mechanism for common measurement of the impact of primary care innovations across Canada is lacking. Currently, pan-Canadian measurement and reporting has been more focused on the acute care sector. For example, a report released on the performance of primary care in Canada in 2016 reported on only 16 of the 51 indicators that policy makers and clinicians had deemed important in 2012 [Canadian Institute for Health Information (CIHI), 2016]. This suggests that little progress has been made toward collecting and reporting primary care indicators; further emphasizing the need for developing common and shared data sources in primary care. More recently Canadian data using the Quality and Costs of Primary Care (QUALICO-PC) indicators have been reported from Canada (Wong *et al*., 2015; Breton *et al*., 2016; Anisimowicz *et al*., 2017; Katz *et al*., 2017).

In order to speed up progress in collecting data using the same measures and data sources across research projects and facilitating knowledge exchange across jurisdictions, the Canadian Institutes of Health Research (CIHR, 2013) and its provincial funding partners required cross-jurisdictional collaboration on indicator measurement and evaluation as part of a signature research initiative on community-based primary health care (CBPHC). The first of its kind in funding structure, the initiative invested $33.4 million in funding to the 12 multi-jurisdictional five-year team grants. These 12 teams are undertaking programmatic, cross-jurisdictional, and interdisciplinary research to develop, implement, evaluate, and compare innovations in primary care. In order to facilitate collaboration across the 12 teams, a condition of this funding was that a portion of the grant money could only be spent on collaboration and cross-team data collection and analysis of common indicators. We report here on the process by which the 12 teams worked together and fostered cross-jurisdictional collaboration, including collection of common indicators with the goal of using the same measures and data sources.

## Methods

### Initial conditions of funding

Twelve CBPHC team grants were selected by a rigorous international peer review process and funded by CIHR (2012) and partners in 2013. The signature initiative’s key research areas included: improving access for vulnerable populations and innovations in chronic disease management and prevention. To encourage building capacity and scaling-up successful innovations, each team was required to include at least two decision-makers from different jurisdictions, at least one health professional, and a community-engagement process to provide feedback on the proposed care delivery model (CIHR, 2012).

Each team was required to allocate $50 000 CAD/year over the course of the grant (total per team=$250 000) for reviewing, agreeing, and operationalizing common measurement and assessment strategies. Although cross-collaboration was among the requirements of receiving the funding, there were no *a priori* decisions on how the teams would work together.

### Working group formation

A working group was established with representatives from each of the 12 teams as well as the funder during the first year of funding. The primary objective of this group was to develop a common set of indicators to describe the knowledge generated by these 12 cross-jurisdictional teams and to develop a process to work across the teams (Riddell *et al*., 2017). The group met through a series of teleconferences and face-to-face meetings, and was chaired by one of the 12 teams’ principal investigators.

### Overarching research questions

The working group agreed upon collecting indicators within several core dimensions of primary care with the intention of addressing three objectives across the 12 teams:

- Describe the impact of improving access to CBPHC, particularly for vulnerable populations;
- Examine the impact of alternative models of chronic disease prevention and management in CBPHC on patient and system outcomes; and
- Describe the structures and context that influence the implementation, delivery, cost, and potential for scale-up of CBPHC innovations.

### Procedures for consensus on indicators and measures

The working group undertook an iterative process of discussion, consideration of existing primary care measures from the literature (Starfield, 2001; Hogg *et al*., 2008; Watson *et al*., 2009) and stakeholder engagement. We incorporated the CIHI (2012) pan-Canadian primary health care indicators and previous work developed in the Canadian context from measurement experts in primary care (Fortin *et al*., 2012; Haggerty *et al*., 2012; Wong *et al*., 2015). Teams were expected to incorporate the agreed upon common measures into their respective programs and to report on them.

In choosing measures and indicators, specific efforts focused on refining them based on feedback from teams’ First Nation and Inuit partners. Team members were asked to reach common ground about the priority dimensions to be included in data collection, where possible, across all of the 12 teams.

### Data collection on their use of the common indicators

Teams were surveyed using self-administered questionnaires on their planned research programs, including study design, methods of data collection, data sources, and study populations, as well as their coverage of the common indicators, and whether they were collecting the exact or modified versions of the indicators.

## Findings

### Description of the teams, their research, and sources of data

The teams were heterogeneous in relation to their specific research questions, structure, and methodology, each with several studies or sub-studies using a variety of methods (Table 1). The teams’ projects were similar in the sense that they were written for internal validity and intervention fidelity with potential for generalizability of results. The teams employed a range of study designs including case studies, cross-sectional observational studies, pre–post intervention, randomized controlled trials, and longitudinal study designs. Taken together, the teams covered all of Canada with the exception of one province and one territory (Saskatchewan, Yukon, respectively) (Table 1). Three teams have Indigenous partners and three are collaborating internationally. Nine of the 12 teams are collecting data in English and French. Some teams are collecting data in other languages, including Cantonese, Greek, and Italian.

**Table 1:** Location and brief description of 12 teams projects

Principal investigators	Project title	Primary location	Other jurisdictions	Collaborating countries
Marshall Godwin Rick Audas, Kate Tilleczek, Scott Ronis, Michael Zhang, Jacques Richard	Barriers and facilitators in access to child/youth mental health services	NL	NB, NS, PE	
Eva Grunfeld	Community-based cancer care along the continuum (CanIMPACT)	ON	AB, BC, MB, NS, QC	
Jeannie Haggerty, Grant Russell	Organizational innovations to improve access to PHC for vulnerable groups	QC	AB, ON	Australia
Stewart Harris	Transformation of Indigenous primary health care delivery (FORGE AHEAD)	ON	MB, NB, NL, QC	
Janusz Kaczorowski	Chronic disease awareness and management program (C-ChAMP)	QC	ON	
Alan Katz, Kathi Avery Kinew Jose Lavoie	Transforming PHC in First Nations and rural/remote communities (iPHIT)	MB	First Nations Inuit Health Branch, Nanaandewewigamig	
Claire Kendall, Clare Liddy	Advancing PHC for persons living with HIV/AIDS	ON	MB, NL	
Jenny Ploeg, Maureen Markle-Reid	Community-based approaches for older adults and their caregivers	ON	AB	
Moira Stewart, Martin Fortin	Patient-centered innovations for persons with multimorbidity (PACE in MM)	ON	QC	
Walter Wodchis, Toni Ashton, Mylaine Breton, Tim Kenealy, Nicolette Sheridan	Implementing integrated care for older adults with complex health needs (iCOACH)	ON	QC	New Zealand
Sabrina Wong, William Hogg, Fred Burge, Sharon Johnson	Transforming community-based PHC through comprehensive measurement and reporting (TRANSFORMATION)	BC	ON, NS	
Kue Young	Transforming PHC in remote northern communities: the circumpolar health system innovation team (CIRCHSIT)	AB	NL, NU, NW	Finland, Sweden, Alaska

NL=Newfoundland; NB=New Brunswick; NS=Nova Scotia; PE=Prince Edward Island; ON=Ontario; AB=Alberta; BC=British Columbia; MB=Manitoba; QC=Quebec; PHC=primary health care; NU=Nunavut; NW=Northwest Territories. More information about the projects can be found at http://www.cihr-irsc.gc.ca/e/45817.html

### The dimensions of primary care and the agreed upon common indicators

Based on the funded teams’ proposals and contexts, feasibility, collective expertise, and primary health care indicators developed by the CIHI (2012), the working group agreed upon five primary health care dimensions, namely, access, comprehensiveness, coordination, effectiveness, and equity (see Table 2).

**Table 2:** Coverage of agreed upon primary care indicators across the 12 teams

Dimensions	Indicator	Teams collecting exact or modified item	Data source
*Access*: ability to access routine or ongoing PHC	Difficulties accessing routine or ongoing PHC	12	Pa survey
*Comprehensiveness*: the Provision, either directly or indirectly, of a full range of services to meet patient’s health care needs	Scope of PHC services: patient survey	8	Pa survey
	Scope of PHC services: clinician survey	6	Pr survey
	Scope of PHC services: org attributes	8	Org survey
*Coordination*: facilitating patient care between organizations	PHC team effectiveness	8	Org survey
	Collaborative care with other health care orgs	10	Org survey
	Information management and continuity	10	Pa survey Org survey HAD
*Effectiveness*: the extent to which the outputs of the organization, make a positive contribution to the health and wellness of patients	Self-efficacy managing chronic diseases	7	Pa survey
	Patient centeredness	8	Pa survey
	Patient empowerment	7	Pa survey
	Hospitalizations for ambulatory care sensitive conditions	7	HAD
*Equity*: ensuring access to health care and to quality services on the basis of health needs	Vertical	10	Pa survey
	Horizontal	9	Pa survey HAD
*Other indicators*: health care costs	Costs associated with use	6	Pa survey HAD
	Community care costs	6	Pa survey
	Funding arrangements	6	Pr survey
	Patient out of pocket costs	6	Pa survey
*Other indicators*: patient health, and well-being	Health status (EQ-5D-5L)	7	Pa survey
	Health and well-being (VR-12)	9	Pa survey

PHC=primary health care; Pa=patient; Pr=clinician; Org=organization; HAD=health administrative data.

**Figure 1:**
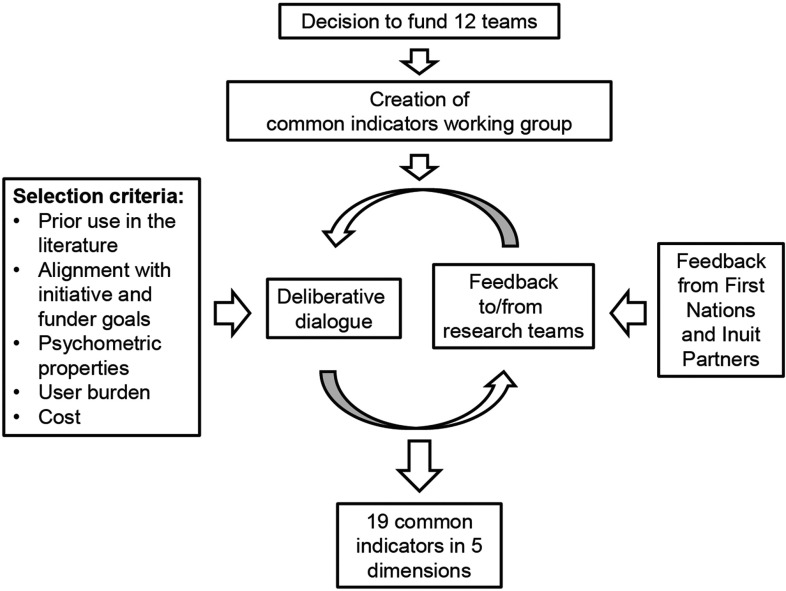
The process and criteria of development of the common indicators among the 12 teams

We identified 19 indicators covering the five dimensions that could be measured using publicly available items and scales (see Additional File 1). Common indicators in addition to those within the five dimensions include population/patient characteristics, multimorbidity, patient-reported outcome measures (PROMs) of health and well-being [VR-12-SF (Ware *et al*., 1996) and EQ-5D-5L (Xie *et al*., 2016)], health care utilization, and health care costs. All scales, including the Team Climate Inventory (Anderson and West, 1998) were selected based on having appropriate reliability and validity, prior use in primary care, user burden (where possible, items that were already being collected by some teams or could be easily added to planned data collection) and cost (with a preference for tools that are available free of charge to facilitate sustainability of this work) (Figure 1).

Data sources included patient, provider, and organizational surveys, and common algorithms in health administrative data. Validated tools for use with administrative health data include common algorithms for identifying age strata or examining multimorbidity, as examples.

Methods used to collect the data included interviews or secondary analysis of health administrative data after an approved data access request was completed (five of these teams are linking these data with other sources such as survey data) (Table 3).

**Table 3:** Sources and types of data collection used by the 12 teams

Type of data source	Teams collecting data
*Quantitative*	
Patient survey	8
Clinician survey	5
Organizational survey	6
Team Climate Inventory	7
Electronic medical records	4
Health administrative data	9
HAD linkage to other data sources	5
*Semi-quantitative*	
Manual chart review	2
*Qualitative*	
Focus groups	7
Semi-structured key informant interviews	9
Case studies	5
Other qualitative data	3
Other data^a^	3

a Other data collected by teams include items such as community readiness surveys, volunteer satisfaction surveys, and publically available statistical data.

### Coverage of indicators by the teams and sources of data

Some teams had started collecting data prior to the funding decision and formation of the working group. Where possible, the agreed upon measures were incorporated into teams’ research plans after the funding decisions were made and the working group was formed. Some teams are collecting modified versions of the agreed measures.

The most commonly collected indicators are in the dimensions of access (all teams), coordination (11/12 teams), and equity (11/12), whereas the least commonly collected indicators are related to health care costs (six teams) (Table 2). In addition, all teams are collecting measures related to patient characteristics, multimorbidity, and health care utilization.

### Group process for cross-team collaboration

Following collaboration on the common indicators, the working group devised an authorship policy ratified by the principal investigators of all teams. This policy is informed by previous policies made by some of the 12 teams and their institutes as well as academic publishing guidelines [Hogg *et al*., 2014; International Committee of Medical Journal Editors (ICMJE), 2017].

The working group is now developing and implementing cross-team collaborative projects using data collected through the common indicators. Cross-team project ideas are pitched to the group in the form of one-pager summaries and smaller working groups are formed to lead the studies. Progress is shared with the larger group and other researchers can join at different stages. Cross-team projects are facilitated with the help of a cross-team research coordinator.

This work provides the largest pan-Canadian opportunity for cross-jurisdictional learning and knowledge generation on the processes, outputs, and patient-reported outcomes in primary care service delivery. For example, across the teams, we now have the largest source of data on team functioning in primary care practices and the largest source of data on PROMs in the Canadian primary care context.

## Discussion

This unprecedented process of collaboration across 12 interdisciplinary CBPHC research teams operating across Canada in 13 jurisdictions is a unique opportunity for new knowledge generation that exceeds beyond the knowledge outputs of any one team. We have successfully built the infrastructure and fostered partnerships across the teams to advance primary care research and innovation. Across the teams, there are over 120 individual researchers, staff, and students who have developed ways for working together across Canada and now share common ways of measuring key dimensions of primary care.

The collected common indicators can serve as a standard set of items and scales for common primary care performance reporting across Canada; they could complement the gaps in data for existing reporting initiatives such as reporting on the CIHI (2016) pan-Canadian primary care indicator set. As an added value to such quantitative measures, our combined efforts provide a richness of multiple data sources, diverse perspectives, and contextual factors. Finally, cross-comparative analysis of data collected through validated tools such as EQ-5D-5L and Team Climate Inventory in different contexts could inform of their utility in the Canadian primary care.

In the context of research impact (Greenhalgh *et al*., 2016), the development of common metrics, a data infrastructure, and a collaborative process across the 12 teams has the potential to make a dramatic contribution to the key aims of the signature initiative. In addition, because of the heterogeneity of the teams and the diversity of local context, the learnings of the 12 teams and the working group bring more real world relevance to measurement of primary care. The data collected through the common indicators, complemented with local context, can answer questions such as ‘what works for whom in what circumstances’ (Pawson and Tilley, 2001). The working group across the teams is an innovation in how to best share and reflect on heterogeneous and common data to foster improvements in primary care delivery and organization. Moreover, we have developed capacity across these teams to collect data for future analysis and bring understanding of the context about outcomes related to spread and scale of innovations in delivery and organization of primary care.

A key factor in the success of the common indicators working group is each team’s willingness to collaborate openly, engage with each other and commitment to the process. Other key success factors include leadership of the working group that entailed active engagement of all members, a research coordinator, and regular liaising with the funder and different teams based on their needs. An additional condition needed for this work is embedded funding. By allocating funding and mandating cross-team cooperation, CIHR and its partners has created an environment for collaboration rather than competition.

This work began with broad aims and varied methodologies, which required dedicated collaboration on behalf of the research teams in order to establish common ground and move towards the cross-team objectives (CIHR, 2012). The lack of standardization in applying the measures and data collection will limit the kinds of analyses possible. Future analyses will require a thorough assessment of potential bias introduced by the heterogeneity of data collection methods. At the very least, we now have a foundation of researchers and trainees from which to build cross-team research and can incorporate important learnings from other examples of cross-team research in primary care, such as the Agency for Health care Research and Quality Patient-Centred Medical Home research wherein specific goals and objectives were defined at the beginning and projects were carried out independently, with outcomes pooled at the end (Genevro and Meyers, 2013).

Reaching consensus on common indicators was challenging at times. In part, this was a normal process of group formation where we moved through the norming, storming, and forming stages (Tuckman, 1965). Moreover, there has been turnover of investigators and staff in this group. Much of our disagreement was influenced by the high value placed on traditional approaches to research methods. These methods are guided by fidelity to *a priori* specification of hypotheses-testing, which drives sampling, randomization, and data collection processes.

An ongoing challenge is having the working group members act as liaisons between the working group and their individual teams, the latter of which include community partners and other diverse stakeholder groups. Finding ways to optimize the group process and utilize all expertise (funders, researchers, and community partners) to develop a mutually acceptable set of measures and common approach to data collection are resource and time intensive. Indeed, each team found it important to respect the values and preferences of their represented sub-populations while also collaborating with other teams and moving toward common goals.

Other challenges associated with conducting cross-team research include confidentiality related to data sharing and ensuring accurate and meaningful comparisons of results across teams with divergent study designs, target patient populations and that not all teams were using methods compatible with the entire set of measures. Ownership of data is of critical importance in relation to working with First Nations and Inuit partners; teams who were specifically committed to participatory research methods and principles of *Ownership*, *Control*, *Access*, *Possession* (First Nations Information Governance Centre, 2014) meant that decisions to collect common indicators and participate in cross-team analysis of data require additional memoranda of understanding with Indigenous community partners. In that regard, the data-sharing processes opted by the common indicators working group are experiments that can be replicated in other contexts to further facilitate cross-jurisdictional and collaborative PHC research.

## Conclusions

Twelve CBPHC research teams, supported by specific funding, formed a working group and engaged in a process to agree on and collect common data indicators for the generation of knowledge beyond any one team’s ability. As a result of this signature initiative, a wealth of data stemming from the common indicators has been generated in primary health care. Collected process, outcome, and contextual data from the 12 teams will be analyzed using mixed methods to describe elements of the interventions and the contexts that will influence their implementation and scale-up. Future work should define measures *a priori* within request for proposals and require that teams build this into their applications. Such an approach could enable funders and researchers to synthesize findings across funded studies to be able to give a cohesive picture of findings from an initiative.

The 12 teams collaboration has laid the groundwork for measurement in future CBPHC endeavors, and while the measures may not be directly transferable to other high-priority areas of health care (eg, end-of-life care) our framework and processes can be used to inspire and fast-track similar future initiatives. Our findings can provide guidance for those at the coalface of innovation in primary care from practice-level service delivery and planning through to national performance measurement and reporting.
